# Supplementary figures and images for: Isorhamnetin and Hispidulin from Tamarix ramosissima Inhibit 2-Amino-1-Methyl-6-Phenylimidazo[4,5-b]Pyridine (PhIP) Formation by Trapping Phenylacetaldehyde as a Key Mechanism
Source: Foods. 2020 Apr 3;9(4):420. doi: 10.3390/foods9040420 (PMC7230572; doi:10.3390/foods9040420)

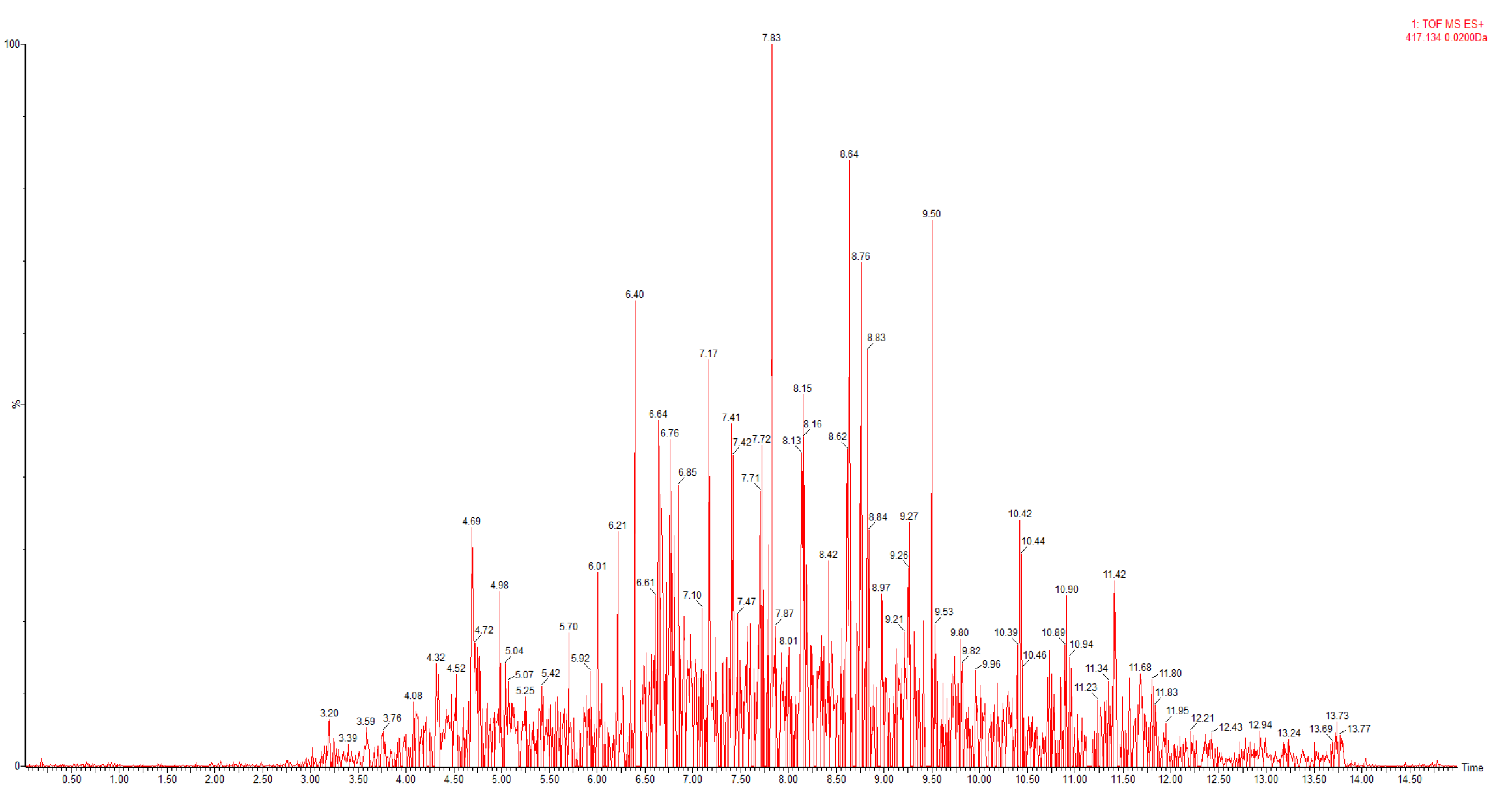

Supplement: Supplementary file 1 [file foods-09-00420-s001.zip › foods-725849-supplementary Figure S1.tif]
